# Unlocking the Diagnostic Potential of Saliva: A Comprehensive Review of Infrared Spectroscopy and Its Applications in Salivary Analysis

**DOI:** 10.3390/jpm13060907

**Published:** 2023-05-28

**Authors:** Charlotte Delrue, Sander De Bruyne, Marijn M. Speeckaert

**Affiliations:** 1 Department of Nephrology, Ghent University Hospital, 9000 Ghent, Belgium; charlotte.delrue@ugent.be; 2 Department of Clinical Biology, Ghent University Hospital, 9000 Ghent, Belgium; sander.debruyne@uzgent.be; 3 Research Foundation-Flanders (FWO), 1000 Brussels, Belgium

**Keywords:** infrared spectroscopy, saliva, non-invasive biomarker

## Abstract

Infrared (IR) spectroscopy is a noninvasive and rapid analytical technique that provides information on the chemical composition, structure, and conformation of biomolecules in saliva. This technique has been widely used to analyze salivary biomolecules, owing to its label-free advantages. Saliva contains a complex mixture of biomolecules including water, electrolytes, lipids, carbohydrates, proteins, and nucleic acids which are potential biomarkers for several diseases. IR spectroscopy has shown great promise for the diagnosis and monitoring of diseases such as dental caries, periodontitis, infectious diseases, cancer, diabetes mellitus, and chronic kidney disease, as well as for drug monitoring. Recent advancements in IR spectroscopy, such as Fourier-transform infrared (FTIR) spectroscopy and attenuated total reflectance (ATR) spectroscopy, have further enhanced its utility in salivary analysis. FTIR spectroscopy enables the collection of a complete IR spectrum of the sample, whereas ATR spectroscopy enables the analysis of samples in their native form, without the need for sample preparation. With the development of standardized protocols for sample collection and analysis and further advancements in IR spectroscopy, the potential for salivary diagnostics using IR spectroscopy is vast.

## 1. Introduction

**Figure 1:**
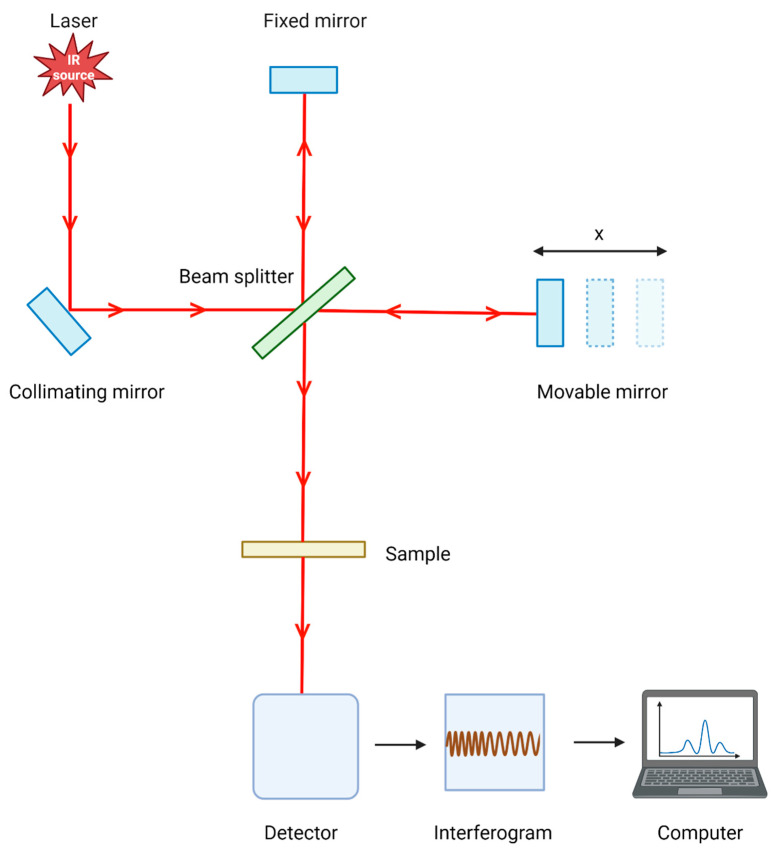
The general principle of Fourier-transform infrared spectroscopy. The radiation from a polychromatic source is captured and made parallel using a collimating mirror. These parallel rays collide in a beam splitter and are split into two beams: one reflected by a stationary mirror and the other reflected by a moving mirror. Depending on the distance between the static and moving mirrors, the recombined reflections of the beam at each mirror can result in either constructive or destructive interference. This procedure results in two beams once more, one of which travels back to the power source, and the other is directed at the sample and detected by the detector. An interferogram of this data is built up in the memory of the computer. Hundreds or thousands of interferograms can be accumulated due to the process’ rapidity (often less than 1 s) and excellent precision. These interferograms can then be combined and translated using Fourier transform to create standard transmittance (or absorbance) spectra against wavenumbers.

Traditional methods for collecting diagnostic samples, such as blood or tissue biopsies, are invasive, uncomfortable, and time-consuming. Additionally, they may require specialized equipment and trained personnel for collection and analysis. These limitations can lead to delays in diagnosis and treatment, which can be detrimental to patient outcomes. Therefore, there is a need for non-invasive and rapid diagnostic methods that can provide accurate and reliable results. Saliva is an important diagnostic fluid that is readily available for non-invasive collection, making it an ideal sample type for disease detection and monitoring. It contains various biomolecules such as lipids, carbohydrates, proteins, and nucleic acids, which can provide valuable information about an individual’s health status. Moreover, saliva-based diagnostics can be particularly useful in settings in which traditional diagnostic methods, such as remote or resource-limited areas, are not readily available or feasible. Currently, enzymatic, colorimetric, chromatographic, kinetic, and mass spectrometric analyses, as well as immunoassays, are used to analyze saliva [1]. In recent years, various novel techniques including vibrational spectroscopy and biosensors have been developed for the analysis of salivary biomolecules. Implementing Fourier-transform infrared (FTIR) spectroscopy (Figure 1) for diagnosis and treatment of diseases involves a series of steps [2]. First, saliva samples are collected from individuals suspected of having the disease or from a representative population. The samples are then prepared by removing the debris through centrifugation or filtration. Next, an FTIR spectrometer capable of collecting high-quality infrared (IR) spectra is acquired. The instrument should be calibrated using appropriate reference standards to establish a baseline for spectral measurements. FTIR spectra are collected from the prepared saliva samples, aiming to capture variations associated with the disease. Preprocessing techniques are applied to enhance the quality of the data, such as improving the signal-to-noise ratio and eliminating instrumental artifacts or background noise. The preprocessed FTIR spectra are analyzed to extract relevant information. This analysis includes comparing spectra from different disease stages, identifying characteristic peaks indicative of, e.g., viral alterations, and quantifying the concentrations of multiple infections present in saliva samples. Statistical analysis techniques, such as multivariate analysis, clustering algorithms, or classification models, are employed to derive insights from the FTIR data. These techniques help to differentiate between disease stages and identify specific infections. To validate the FTIR-based disease tracking and detection method, the results are compared with established diagnostic methods using appropriate reference standards or gold standard tests. Continuous optimization and refinement of the FTIR protocol are important. Feedback and further research are considered, and advanced data analysis techniques, such as machine learning algorithms, may be explored to enhance disease tracking and identification capabilities. Collaboration with experts in spectroscopy, biology, and medical professionals is essential to ensure the accuracy and clinical relevance of the results. 

The present review discusses IR spectroscopy-based analytical methods for saliva, which offer a promising avenue for non-invasive and rapid diagnosis of various diseases. Salivary biomarkers have been identified for diseases, such as dental caries, periodontitis, infectious diseases, cancer, diabetes mellitus, and chronic kidney disease, as well as for drug monitoring. 

## 2. Methodology 

This narrative review is based on a literature search of the online PubMed Database. The keywords (“saliva” [MeSH Terms] OR “saliva” [All Fields] OR “salivas” [All Fields] OR “salivas” [All Fields]) AND “infrared” [All Fields] AND (“spectroscopies” [All Fields] OR “spectroscopys” [All Fields] OR “spectrum analysis” [MeSH Terms] OR (“spectrum” [All Fields] AND “analysis” [All Fields]) OR “spectrum analysis” [All Fields] OR “spectroscopy” [All Fields]) AND (“medicin” [All Fields] OR “medicinal” [All Fields] OR “medicinally” [All Fields] OR “medicinals” [All Fields] OR “medicine” [MeSH Terms] OR “medicine” [All Fields] OR “medicines” [All Fields] OR “medicines” [All Fields]) yielded 74 results. This search was limited to papers published in English, articles/reviews from the last 15 years, and articles with available attached files. Following this selection, another narrowing down of the papers was made based on the full text according to the following inclusion and exclusion criteria. The criteria for selecting the subjects were as follows:

- Inclusion criteria:
  o Publication year: 2008–2023
  o Language: English
  o Study design: cross-sectional, case-control, and cohort studies
  o Participants: humans and animals
  o Sample: saliva
  o Technique: all types of infrared spectroscopy
- Exclusion criteria:
  o Publication year: older than 2008
  o Language: Non English
  o Study design: no restriction
  o Participants: no restriction
  o Sample: blood, serum, urine (except in combination with saliva)
  o Technique: other spectroscopic techniques (Raman spectroscopy and mass spectroscopy)

The references for the final selection of papers were also analyzed for relevance following the inclusion and exclusion criteria described above. 

## 3. Unlocking the Secrets of Saliva: A Comprehensive Analysis of its Complex Composition 

On an average day, a healthy adult generates approximately 600 mL of saliva, which typically has a pH level that falls within the range of 6.6 to 7.1 [3]. Saliva, a crucial fluid for oral health and food digestion, is produced by the salivary glands in the mouth. It is a complex mixture of biomolecules such as water, electrolytes, proteins, lipids, carbohydrates, and nucleic acids. Water is the main component of saliva, accounting for approximately 99% of its volume. The remaining 1% consists of various electrolytes, such as potassium, sodium, chloride, phosphate, and bicarbonate, which help to maintain the pH and osmolarity of saliva, mucus, proteins/peptides, enzymes, nucleic acids, and hormones. Salivary proteins are classified into two main categories: secretory and non-secretory. Secretory proteins, such as mucins, amylase, and proline-rich proteins, are synthesized and secreted by salivary glands and play important roles in lubrication, protection, and digestion. Non-secretory proteins such as immunoglobulins and enzymes are derived from plasma and transported into saliva through a process called transcytosis [4,5]. Thus, salivary biomarkers can be used for the early detection of certain systemic diseases [3,6,7,8]. Lipids such as phospholipids, glycolipids, and cholesterol are also present in saliva, even at relatively low concentrations. These lipids may play a role in the maintenance of oral health and prevention of bacterial colonization. Carbohydrates, such as glucose and fructose, are present in small amounts in saliva and are derived from diet. Salivary nucleic acids such as RNA and DNA are also present, although their functions in saliva are not yet fully understood. Saliva collection is simple, fast, and safe. Collecting saliva is less painful for patients and requires less handling during diagnostic procedures than blood collection [7,9,10]. Saliva content is not uniform and undergoes modifications based on factors such as the level of gland stimulation, time of day, dietary intake, and overall health [11].

## 4. Illuminating the Chemical Makeup of Saliva: Exploring the Relationship between Infrared Spectroscopy Techniques and Salivary Composition 

IR spectroscopy is a powerful technique that has been widely used to analyze various biological samples, including saliva. This technique uses the measurement of the absorption, transmission, or reflection of IR radiation by the molecular vibrations of the sample. IR spectra convey both quantitative and qualitative information about the nature, structure, and molecular environment of chemical bonds. While the intensities of the IR spectra provide quantitative data, the frequencies indicate qualitative characteristics. The collective contributions of these aspects comprise an IR spectrum, which serves as a molecular fingerprint and captures all pathological changes in cells, tissues, or fluids. IR spectroscopy provides information on the chemical composition, structure, and conformation of biomolecules in a sample and can be used for diagnostic purposes [12]. Conventionally, the IR region is divided into three regions: far-infrared (<400 cm^−^^1^), mid-infrared (MIR, 4000–400 cm^−^^1^), and near-infrared (NIR, 13,000–4000 cm^−^^1^) [13]. NIR utilizes endogenous chromophores, such as oxy- or deoxyhemoglobin, lipid or water bands, or a combination of these diagnostic markers, to detect differences [14]. The most significant vibrations observed in biological samples are attributed to carbohydrates and nucleic acids (1225 cm^−^^1^), lipids (1750 cm^−^^1^), and proteins (amide I: 1550–1600 cm^−^^1^ and amide II: 1600–1700 cm^−^^1^). [15]. The displacement of spectral bands and strength of spectral modes are useful spectral parameters that provide significant information about the composition of the sample. This information can be utilized for monitoring and diagnosing a variety of diseases [16].

Several IR spectroscopy techniques are available, including attenuated total reflectance (ATR), FTIR, and micro-attenuated total reflectance (micro-ATR) spectroscopy. ATR-FTIR spectroscopy is a highly sensitive, globally applicable, and reproducible physicochemical analytical method that utilizes IR absorption to identify structural molecules [17]. The ATR-FTIR spectrum of a biomolecule is determined by the specific structural bonds present in the molecule, which are unique to that particular biomolecule [16,17]. The sample is placed in a single cell and exposed to IR radiation to obtain its absorption spectrum. ATR-FTIR spectroscopy is becoming increasingly popular for distinguishing various chronic diseases because of its numerous advantages, such as being non-invasive, label-free, quick, high-capacity, economical, and offering comprehensive details on the chemical structure and molecular makeup of specimens [18,19]. The sample is placed on a diamond or germanium crystal, and IR radiation is passed through the crystal. ATR detects the amount of IR radiation absorbed by a sample as it interacts with the crystal. Micro-ATR spectroscopy is a variation of ATR spectroscopy, and is used for the analysis of small samples. This technique allows the collection of spectra from microscopic areas of the sample, enabling the analysis of small volumes and localized regions of interest. FTIR, ATR, and micro-ATR spectroscopy are powerful techniques that offer advantages such as being non-destructive, label-free, and rapid. In biological specimens such as saliva, the IR spectral modes can be associated with biochemical signatures that are directly related to the presence or absence of diseases. Additionally, these modes form the foundation for quantitatively measuring several analytes, making them vital for both monitoring and diagnostic purposes [12,20].

**Figure 2:**
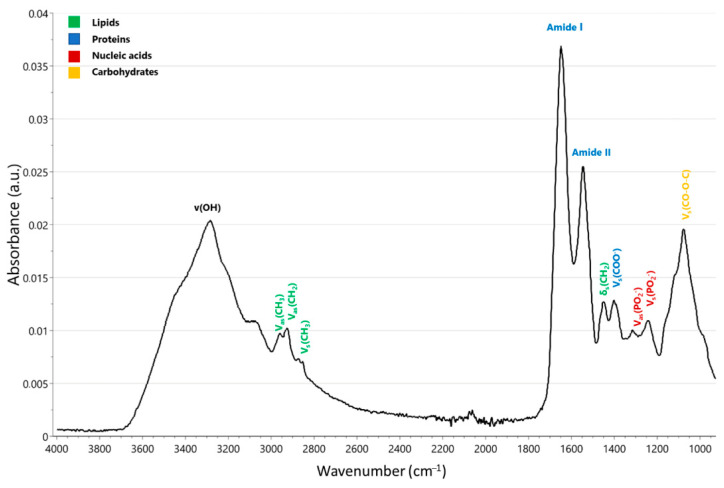
A typical MIR spectrum measured with attenuated total reflection-Fourier transform infrared (ATR-FTIR) spectroscopy of a saliva sample showing peak assignments from 4000–800 cm^−1^. V: stretching vibrations, δ: bending vibrations, s: symmetric vibrations, as: asymmetric vibrations.

The characteristic IR spectrum of saliva reveals the presence of three primary categories of macromolecules, including lipids (3000–2800 cm^−^^1^), proteins (1700–1600 and 1560–1500 cm^−^^1^), and nucleic acids (1250–1000 cm^−^^1^) (Figure 2). The amide A band, characterized by a broad peak at 3273 cm^−^^1^ and a medium-intensity narrow peak at 2057 cm^−^^1^, corresponds to thiocyanate (SCN^−^) anions and is characteristic of saliva. The bands located at 1649 and 1543 cm^−^^1^ are identified as amide I and II, respectively, while the band observed at 1075 cm^−^^1^ can be attributed to sugar fragments. The remaining absorption bands, which are relatively intense, can be assigned to the methylene groups present in the side chains of amino acids in proteins and lipids (1452 cm^−^^1^), the side chains of amino acids (1396 cm^−^^1^), amide III/phospholipids (1286–1320 cm^−^^1^), and various fragments of sugars, glycosylated proteins, and nucleic acid phosphate groups (1080–950 cm^−^^1^). The bands detected at 1080–950 cm^−^^1^ can be linked to various sugar residues present in saliva, including glycosylated α-amidase. When a standard IR spectrum of saliva was compared to those of albumin, glucose, and lysozyme, similarities were observed in the 1700–1400 cm^−^^1^ range with the albumin spectrum and in the 1100–1040 cm^−^^1^ range with the glucose and lysozyme spectra [21]. 

A study conducted to validate this concept showed the potential to identify distinct sources of variability that influence the IR data obtained from salivary samples. Tobacco smoking and gender were also investigated. Patients were grouped into three distinct classifications based on their smoking habits: non-smokers, social smokers, and regular smokers. Various bands were found to be distinguishing factors among the different groups of smokers, notably within the wavenumber range of 1950–2150 cm^−^^1^ (with a peak around 2060 cm^−^^1^, corresponding to thiocyanates). Additionally, some more scattered bands were found to differentiate tobacco, including the amide I band (mainly stretching of C=O) and the amide II band (mainly bending of N-H), which were situated in the spectral range of 1500–1700 cm^−^^1^ [22]. The increase in the level of thiocyanates in saliva could be associated with a higher frequency and duration of smoking [23]. Another study examined the impact of smoking cessation on the composition of salivary compounds using FTIR spectroscopy. Despite the small differences in spectral intensity observed between the two groups, the smoker group exhibited a slight increase in spectral peaks, particularly in the bands associated with DNA, indicating a possible modification of its content or cell necrosis. Additionally, mannose-6-phosphate had elevated spectral peaks in the smoker group. In contrast, in the group of former smokers, the peak for SCN- decreased while the intensity of the collagen band increased, indicating a better tissue regeneration capacity in individuals who have ceased smoking [24]. Wavenumbers between 1735 and 1740 cm^−^^1^, which are associated with the C=O esters of lipids, indicate sex differences [22]. A different study corroborated this finding by showing that ATR-FTIR spectroscopy can identify the gender of fertilized unincubated chicken eggs by detecting the symmetric stretching vibration of fatty acid esters around 1740 cm^−^^1^, which is crucial for this process [25]. The discriminant bands were utilized to apply linear discriminant analysis (LDA), resulting in the construction of a classifier that displayed strong predictive capability. The classification rates achieved for females and males were 81.6% and 82.4%, respectively. The biochemical composition of saliva can also be influenced by various other factors, such as oral medication, stress, age, body mass index (BMI), and comorbidities associated with complex diseases [22].

To examine the unique differences and connections between the MIR spectra of saliva and oral physiology and behavior (such as saliva flow, fungiform papillae, and oral processing time), a study involving 52 participants was conducted to analyze and interpret these factors. Unstimulated saliva samples were collected from these participants, and their MIR spectra were analyzed to develop partial least squares (PLS) regression models. The R^2^ coefficient of determination and the standard error in cross-validation (SECV) were calculated for saliva flow rate (R^2^: 0.65–0.83, SECV: 0.25–0.31), papillae density of tongue (R^2^: 0.73–0.83, SECV: 7.4–11.2), and oral processing time (R^2^: 0.7–0.87, SECV: 1.66–3.04). Optimal models were obtained when participants were divided into groups based on age and sex. It is important to mention that diverse PLS loadings were identified in the MIR fingerprint regions among various demographic groups, implying that different constituents of saliva are linked to variations in oral sensing physiology and oral processing within distinct demographic groups. These results suggest that salivary fingerprint spectra can aid in better understanding the consumer eating experience and food selection, making them a valuable asset for marketing and sensory experiments [26].

## 5. Shining a Light on Saliva-Based Diagnosis: Exploring the Applications of Infrared Spectroscopy

### 5.1. Seeing beyond the Surface: Unraveling the Molecular Changes in Oral Fluid for Enhanced Diagnosis of Dental Caries and Periodontitis Using IR Spectroscopy

A crucial approach in healthcare is the implementation of precise monitoring methods for early disease prevention. The primary emphasis is on socially significant illnesses, with dental caries-related diseases being of particular importance because of their significant impact on people’s social lives [27]. Alterations in salivary molecular makeup linked to the development of dental caries can serve as effective tissue markers for tracking the progression of this condition [28]. It is possible to gain novel insights into the progression of dental caries by analyzing changes in the salivary molecular composition at various stages of oral pathology using FTIR (Table 1). The comparison of various features in IR spectra, as well as the determination of the mineral-organic ratio, the carbon-phosphate ratio, the Amide II/Amide I ratio, and the protein/thiocyanate ratio in oral fluid from individuals with and without multiple caries was made possible through the use of FTIR, including synchrotron radiation [mineral-organic ratio: ratio of the integral intensity of the phosphate bands in the spectral ranges of 1078–900 cm^−^^1^ to the integral intensity of vibration band 1700–1590 cm^−^^1^ associated with Amide I; carbon-phosphate ratio: ratio of the C=O and CH_2_/CH_3_ bonds localized in the range of 1430–1360 cm^−^^1^ to the integral intensity of phosphate bands in the region of 1078–900 cm^−^^1^; Amide II/Amide I ratio: C-N stretching, N-H bending vibrations in the range of 1590–1505 cm^−^^1^/C=O stretching in the interval of 1723–1590 cm^−^^1^; protein/thiocyanate ratio: ratio of the integral intensities of the amide bands (Amide I and Amide II) in the range of 1700–1500 cm^−^^1^ to the integral intensity of −N=C=S vibration bands, arranged at 2150–1950 cm^−^^1^, associated with thiocyanate] (Table 1). A comprehensive analysis of the experimental data revealed that in individuals with multiple caries, there was a shift in the organic–mineral balance in their oral fluid, which led to a reduction in mineral mixtures and an increase in the carbon-based component. The increase in the Amide II/Amide I ratio was a crucial factor in these changes in molecular composition, with the group with multiple caries showing a 120% increase compared to those without. This suggests that changes in the composition of the organic component, relative to the proportion of C=O bonds, are caused by an increase in the number of C-N and N-H molecular groups in the oral fluid [29]. These molecular units are linked to protein elements, and changes in their concentration can occur owing to the appearance of pathological microflora in the buccal cavity, as observed in both non-stimulated and stimulated saliva [28,30,31]. The presence of SCN-, as observed in the IR spectrum at 2150–1950 cm^−^^1^, which doubled, was the most prominent indicator of changes in the composition of oral fluid in individuals with multiple caries [29]. 

During pathological processes in humans, the level of SCN- in saliva, which possesses antibacterial properties, can be increased [32]. Although thiocyanate is an important marker of saliva and an antimicrobial agent, it is insufficient for the in vivo diagnosis of future caries because it is only weakly associated with dental caries. Thus, an all-inclusive examination that takes into account both quantitative and qualitative information on the molecular makeup of oral fluid has the possibility to enhance the precision of detecting forthcoming carious processes and boost the preventative diagnosis of this ailment. Additionally, the presence of carboxyl groups in esters, lipids, and carbohydrates (IR spectra within the range of 1765–1725 cm^−^^1^) in mixed saliva is characteristic of caries development. The characteristics of the IR spectra of oral fluid described earlier, along with the shifts in molecular composition indicated by the ratios, suggest that people with multiple cavities have an altered organic–mineral balance in their oral fluid. This leads to a decrease in the presence of mineral groups and complexes and an increase in the organic component [29].

Periodontitis is a common oral disease that affects the supporting structures of teeth, including the gums, periodontal ligament, and alveolar bone. This disease is characterized by inflammation and tissue destruction around the teeth, which can lead to tooth loss if left untreated. Early detection and diagnosis of periodontitis are essential for effective treatment and prevention of further damage. IR spectroscopy has emerged as a valuable tool for diagnosing and monitoring periodontitis. Studies have shown that IR spectroscopy can be used to detect changes in the concentration and composition of salivary biomolecules in patients with periodontitis. A cross-sectional study was conducted to evaluate the capability of infrared attenuated total reflection (IR-ATR) spectroscopy to identify variations in the composition of saliva supernatants between individuals without periodontitis and those with generalized aggressive periodontitis. The prominent variation in absorbance between the two groups was observed within the spectral range of 1230–1180 cm^−^^1^. The observed absorption variation in the region around 1200 cm^−^^1^ could be attributed to the overlapping wings of the two robust PO_2_^−^ stretching vibration bands, or it could be due to an unassigned feature. Principal component analysis (PCA) and analysis of variance (ANOVA) confirmed a significant distinction (as high as 99.8% based on PCA) in the spectral profiles of saliva supernatants between the control group and patients with generalized aggressive periodontitis [33]. In other studies, it was possible to distinguish between patients with gingivitis and periodontitis and those with a healthy periodontium by analyzing the lipid and protein content in the discriminant wavenumber range of 2800–3000 cm^−^^1^ (CH_2_ and CH_3_ stretching vibrations) [34]. It is worth noting that lipid oxidation is significantly elevated in patients with inflammatory conditions such as gingivitis and periodontitis, as indicated by the presence of an olefinic C-H stretching band at approximately 3000 cm^−^^1^. Lipid oxidation is known to increase considerably during periodontal inflammation [35]. Several additional distinguishing bands were detected, such as protein bands (amide I at 1652 cm^−^^1^ and DNA bands at 1713 cm^−^^1^, indicative of base-paired DNA strands) [34]. Notably, the bands at 950 cm^−^^1^ and 1190 cm^−^^1^ distinguished periodontitis [18,22]. The sugar components of glycosylated proteins, including α-amylase (the most prevalent enzyme in saliva), exhibit significant variations in the spectral range of 950–1080 cm^−^^1^ [36]. As the severity of periodontitis increases, the spectral spots shift farther away from those observed in the less infected or healthy patients. 

### 5.2. Saliva Analysis by Infrared Spectroscopy: A Non-Invasive and Accurate Approach for Cancer Diagnosis

Oral cancer is a significant health concern worldwide, with high mortality rates owing to late-stage diagnoses. Early detection and diagnosis of oral cancer are crucial for successful treatment and improved patient outcomes. IR spectroscopy has emerged as a valuable tool for the diagnosis and monitoring of oral cancer (Table 1). It provides detailed information on the chemical composition and structure of salivary biomolecules, which can serve as potential biomarkers for disease. Studies have shown that IR spectroscopy can be used to detect changes in the concentrations and compositions of salivary biomolecules in patients with oral cancer. IR spectroscopy can detect changes in the ratio of protein secondary structures, such as alpha-helices and beta-sheets, which are indicative of tumor progression. IR spectroscopy can also detect changes in the concentration of nucleic acids such as DNA and RNA, which are indicative of cell proliferation and turnover. Furthermore, IR spectroscopy can provide real-time monitoring of disease progression and treatment responses. Changes in the chemical composition and structure of salivary biomolecules can be detected immediately after treatment, providing important information on the effectiveness of the treatment and the need for further intervention. To determine the diagnostic potential of salivary exosomes, a cross-sectional investigation was performed on the FTIR spectra of salivary exosomes from patients with oral cancer (OC) and healthy individuals. Nucleic acids at 1072 cm^−^^1^, membrane lipids at 2924 and 2854 cm^−^^1^, and transmembrane proteins at 1543 cm^−^^1^ were consistently altered in the IR spectra of OC patients compared to controls. With a sensitivity of 100%, specificity of 89%, and accuracy of 95%, the PCA-LDA discrimination model successfully identified the data, whereas the support vector machine (SVM) displayed 100% training accuracy and 89% cross-validation accuracy [37].

The ATR-FTIR spectra comparison of saliva samples between healthy control subjects and patients with salivary gland tumors revealed that the most noticeable changes manifested in the region ranging from approximately 900 to 1300 cm^−1^, which is regarded as a highly diagnostic region for studying various cancer types [38]. The band observed at approximately 1078 cm^−1^ corresponds to the stretching of both asymmetric and symmetric PO_2_^−^ groups, which originate from inorganic phosphates [39] and the phosphate group present in phospholipids [40]. Studies have demonstrated that this specific spectral characteristic is associated with the involvement of phosphates in various diseases [41]. In addition to the previously mentioned bands, vibration arising from the phosphate group was also apparent in the spectra at approximately 1159, 1239, 985, and 936 cm^−1^ [39]. The peaks detected within the 1000–1200 cm^−1^ range could potentially be linked to C-O stretching vibrations from carbohydrates, leading to the suggestion that the bands observed at approximately 1021, 1040, and 1078 cm^−1^ originate from sugar moieties [21,40,42]. Given that a large portion of salivary proteins is glycosylated, it is reasonable to attribute these peaks to the vibrations of glycosylated α-amylase, mucins, or other sugar residues [39,42,43,44]. In the *tumor mixus* spectrum, there was a noticeable increase in the significant enhancement of the spectral signal at ~1119 cm^−1^, which is attributed to the vibrations of carbohydrates’ ν(C–O) and ν(C–O–C), when compared to the non-cancerous spectrum [45,46]. The peak at 1119 cm^−1^ is often regarded as a distinctive feature in the IR spectrum and serves as a spectroscopic indicator of salivary gland tumors [47]. Most of these vibrations exhibit significant enhancement during disease development. There were also notable differences in the secondary structure of the proteins observed between the ATR-FTIR spectra of the control and salivary gland tumor patients. The maximum peak frequency of the α-helix decreased for the *tumor mixus* spectrum (1634–1640 cm^−1^) in comparison to the control group spectrum (1644 cm^−1^), whereas the β-sheet maximum (1543 cm^−^^1^) band frequency increased for the patients with salivary gland tumors [16,42,46,48,49,50,51]. This could be due to changes in the extent of intermolecular hydrogen bonding in the α-helical and β-sheet structures [52]. Additionally, the most intense band at 1648 cm^−1^ attributed to ν(C=O), ν(CN), and δ(NH) vibrations from α-helix upon deconvolution was split into two additional bands at 1664 and 1641 cm^−1^ due to the disordered structure-solvated [νs(C=O)] and unordered structure [ν(C=O)], respectively. The content of α-helical conformation was also significantly lower in *tumor mixus* patients, which could be related to the formation of the β-sheet structure. [53]. It is also noteworthy that the relative intensity of the 1631 cm^−1^ band due to the β-sheet structure [ν(C=O)/ν(C=C)] significantly decreased with cancer development. Conversely, the composition of the 1615 cm^−1^ spectral signal ascribed to the β-sheet formation [ν(C=C)] increased considerably for *tumor mixus* spectral data [16,42,46,48,49,50,51]. Additionally, the peaks observed at ~1403 [νs(COO–), ρb(CH_3_)] and 1450 cm^−1^ [ρb(CH_3_)/δ(CH_2_/CH_3_)] exhibited higher intensities in the *tumor mixus* patients. Other prominent spectral features assigned to proteins can be observed at 1543 [ρb(NH), ν(CN), amide II], 1515 (tyrosine ring, α-amylase, albumin, cystatins, mucins, proline-rich proteins, sIgA), and 1315 cm^−^^1^ [ν(CN), ρb(NH), amide III (α-amylase, albumin, cystatins, mucins, proline-rich proteins, sIgA)]. An additional FTIR signal, which was absent in the control group spectrum, appeared at 1527 cm^−1^ [ρb(NH), ν(C=N), ν(C=C), amide II] [38]. 

According to the World Health Organization’s (WHO) World Cancer Report, breast cancer is the most common and deadly cancer among women worldwide, irrespective of their geographic location or level of development [54]. The rising global incidence of breast cancer, combined with the lack of reliable, cost-effective, and high-throughput detection methods, necessitates the search for alternative diagnostic tools. Therefore, ATR-FTIR spectroscopy has been used to detect breast cancer fingerprints in saliva. The study group consisted of 30 participants: 10 with confirmed breast cancer, 10 with benign breast disease, and 10 controls. The absorbance levels at 1041 cm^−^^1^ in the saliva of breast cancer patients were significantly higher (*p* < 0.05) than those in the saliva of benign patients. Although no significant difference was observed between breast cancer patients and controls, the identified potential biomarker based on spectral analysis showed a remarkable diagnostic value with an AUC of 0.7700, indicating its potential use in breast cancer diagnosis. Using the ROC curve, this resulted in a sensitivity of 80% and specificity of 70% for breast neoplasms vs. control patients, as well as sensitivity of 70% and a specificity of 70% for breast neoplasms vs. benign subjects. The 1041 cm^−^^1^ vibrational mode is caused by higher levels of PO_2_^−^ symmetric stretching [vs. (PO_2_^−^)], which are found in nucleic acids and glycogen. The 1433–1302.9 cm^−^^1^ band area in the saliva of patients with mammary carcinoma was significantly higher (*p* < 0.05) than that in the control and benign patients. The vibrational mode was similar in both the benign and control patients. The area of the salivary ATR-FTIR spectrum has been pre-validated as a potential diagnostic indicator of breast cancer. The AUC of the 1433–1302.9 cm^−1^ salivary band area was 0.835 for breast tumor vs. control and 0.770 for breast tumor vs. benign patients. The higher level in the 1433–1302.9 cm^−^^1^ region is due to the higher levels of COO^−^ symmetric stretching [vs (COO^−^)], which is found in proteins and lipids [55]. Given the higher expression of PO_2_^−^ symmetric stretching [νs. (PO_2_^−^)] and COO^−^ symmetric stretching [νs(COO^−^)] in the saliva of breast tumor patients, these molecules may originate in the blood and enter the saliva via passive diffusion of lipophilic molecules (e.g., steroid hormones) or active transport of proteins via ligand–receptor binding [4]. With a sensitivity and specificity of 90% and 80%, respectively, this spectral biomarker could distinguish human breast neoplasms from controls. Furthermore, with sensitivity and specificity of 90% and 70%, respectively, it was able to distinguish breast cancer from benign disease [55]. As conventional techniques used in clinical practice, such as mammography, ultrasound, and MRI, have sensitivities of 67.8%, 83%, and 94.4%, and specificities of 75%, 34%, and 26.4% [56], respectively, ATR-FTIR spectroscopy has the potential to enhance the precision of breast cancer diagnosis.

### 5.3. Advances in Saliva Analysis: Infrared Spectroscopy as a Promising Technique for Infectious Disease Diagnosis

#### 5.3.1. Catching COVID-19 with Infrared Eyes: How Spectroscopy Is Revolutionizing Diagnosis

Several studies have demonstrated the potential of IR spectroscopy in the detection of infectious diseases through saliva analysis (Table 1). Severe acute respiratory syndrome coronavirus 2 (SARS-CoV-2), responsible for the current global health crisis, has recently become one of the most severe pandemics. Although reverse transcription polymerase chain reaction (RT-qPCR) is widely recognized as the primary diagnostic method, its effectiveness has been compromised by the overwhelming demand for testing. Implementation of this method requires the availability of laboratory resources and chemical reagents. The test typically requires 4–6 h to complete [57]. Diagnostic tests using monoclonal antibodies against SARS-CoV-2 antigens, such as the N protein and S1 or S2 domains of the S protein, can be utilized to detect viral infection through antigen-based testing. However, these tests are less sensitive than RT-PCR [58]. Consequently, vibrational spectroscopy techniques, such as IR spectroscopy, have been suggested as viable options for testing because of their ability to produce reproducible results, require minimal or no sample preparation, and are free of reagents, making them a noninvasive alternative. RT-qPCR analysis was conducted on a set of 237 saliva samples from symptomatic patients, including 138 individuals diagnosed with COVID-19 and 99 without COVID-19. The dataset consisted of MIR spectra, which were assessed using an unsupervised random forest (URF) and classification models. By utilizing both unsupervised and supervised frameworks, it became feasible to effectively emphasize the spectral regions linked to positive samples. These regions include lipids (1785–1729 cm^−^^1^; stretching C=C and C=O of ester groups), proteins (1680 and 1718–1705 cm^−^^1^: stretching C=O and C–N; 1600–1250 cm^−^^1^: amides I, II, and III), and nucleic acids [1612–1606 cm^−^^1^: adenine vibration in DNA; 1244–1100 cm^−^^1^: stretching PO_4_ of phosphodiester groups; 1025–1021 cm^−^^1^: C–O stretching (carbohydrates); 961 cm^−^^1^: deoxyribose; and 930–909 cm^−^^1^: phosphodiester stretching bands]. The individual models demonstrated impressive performances. However, the consensus class achieved even better validation results with 85% accuracy, 93% sensitivity, 83% specificity, and a Matthew’s correlation coefficient of 0.69. The improved performance was attributed to the incorporation of information from various spectral regions. By offering a swift and noninvasive diagnostic technique, this methodology represents a significant resource for reducing expenses and enabling the implementation of risk reduction strategies [57]. 

Additionally, ATR-FTIR spectroscopy has been used to identify unique COVID-19 biological fingerprints, enabling the differentiation between COVID-19-positive and healthy patients. Saliva contains viral particles that are shed from both the upper and lower respiratory tract as well as the salivary glands. Because saliva is in direct contact with both the oral mucosa and salivary glands, which express a high amount of the angiotensin-converting enzyme 2 (ACE2) receptor that binds with SARS-CoV-2, it is considered a reliable biological fluid for testing [59]. Numerous studies have demonstrated that FTIR spectroscopy, particularly when utilizing ATR crystals, can be used to detect this virus [60,61,62,63,64]. A novel approach utilizing ATR-FTIR spectroscopy, which is superfast, reagent-free, and nondestructive, coupled with chemometric analysis, was developed for the prescreening of virus-infected samples. The IR spectra of artificially prepared saliva samples containing inactivated γ-irradiated COVID-19 virus particles were generated at levels as low as 1582 copies/mL, yielding a favorable signal-to-noise ratio. Tentative identification of the primary virus spectral peaks was performed in relation to nucleic acid bands such as RNA. The IR spectral signature of saliva is altered by the presence of viral particles with RNA-associated wavenumbers, which play a key role in discrimination. Discrimination was also achieved through ATR-FTIR spectral analysis of swabs immersed in saliva spiked with varying levels of virus. In a clinical environment with 70 COVID-19-positive and 111 COVID-19-negative swab samples, a sensitivity of 95% and specificity of 89% were achieved. Of the five molecular RNA assignments, four were higher in the negative group than in the positive group. One possible explanation is that the increase in the 1429 cm^−^^1^ region is associated with a virus such as a simple RNA virus. The corresponding decreases in the 1220, 1084, 1069, and 1041 cm^−^^1^ regions may be related to the carrier response to viral infection. The suggested approach is not designed to substitute conventional techniques, such as RT-qPCR; instead, it functions as a fast preliminary screening method [61]. In another study, ATR-FTIR spectroscopy was used to analyze saliva samples obtained from both COVID-19 patients (n = 255) and healthy individuals (n = 1209). Multivariate analysis revealed that the most effective regions for distinguishing between COVID-19 patients and healthy controls were Amide I (1700–1600 cm^−^^1^) and IgG (1560–1464 cm^−^^1^). By utilizing both subregions, the highest metrics of sensitivity (99.2%), specificity (100%), and accuracy (99.6%) were achieved. 

After a detailed analysis of the outcome data produced by the multivariate linear regression model, it was evident that using this model, relying on the vibrations of the Amide I area, yielded better outcomes when applied to a larger sample size. This was due to the fact that the variation in the output values for both the COVID-19 and healthy groups was less than the variations in the IgG region [58]. The underlying pathophysiology of the proposed technology was investigated through controlled infection experiments using Vero E6 cells in vitro and K18-hACE2 mice in vivo to explore its biological basis. In both cell and mouse models, SARS-CoV-2 infection induced additional FTIR signals compared with UV-inactivated SARS-CoV-2 infection. These signals correspond to aggregated proteins and RNA [63]. In comparison to ATR, a new transflection approach resulted in greater absorbance and less noise, resulting in 93% sensitivity and 82% specificity based on the selection of a threshold of 0.6 [64]. 

IR spectroscopy has several applications in personalized medicine for infectious diseases because it provides valuable information about pathogens, host responses, and therapeutic interventions. By analyzing spectral changes in infected tissues or body fluids before and after treatment, clinicians can evaluate the treatment response and make informed decisions about treatment adjustments. This can help personalize treatment regimens and optimize therapeutic outcomes for individual patients. Furthermore, by integrating artificial intelligence and machine learning with IR spectroscopy, we can effectively track disease progression and detect alterations in the chemical composition of viruses based on their unique spectral features. Moreover, this advanced technique enables the identification of the concentration of multiple infections present in saliva [59]. This can be particularly useful in situations in which conventional methods may be time-consuming or limited in their ability to identify emerging or drug-resistant pathogens. It can facilitate timely intervention, reduce reliance on laboratory infrastructure, and improve patient outcomes, particularly in resource-limited settings.

#### 5.3.2. Beyond the Naked Eye: Unveiling the Hidden Clues of Neonatal Sepsis with Infrared Spectroscopy

Neonatal sepsis continues to be a significant global challenge, as it remains one of the leading causes of mortality in newborns. It is estimated that neonatal deaths account for over 40% of deaths among children under the age of five, resulting in the loss of approximately 3.1 million neonatal lives each year worldwide [65]. Despite being the gold standard for diagnosing neonatal sepsis, blood cultures have many limitations [66]. In a prospective cross-sectional study [67], saliva samples were collected from 60 newborns, of which 30 were considered to be at risk of sepsis, while the remaining 30 were healthy newborns serving as the control group. The mean absorbance values at wavenumbers 970, 1037, 1051, 1240, 1301, 1545, and 1640 cm^−^^1^ were lower in the sepsis group than in the control group. The observed difference in absorbance could be attributed to alterations in the salivary biochemical composition of newborns at risk of sepsis. The measured absorbance at specific wavenumbers indicates changes in the DNA and protein composition in the saliva of these newborns, which can be linked to an inflammatory process. This finding is consistent with that of a previous study showing protein damage in neonatal sepsis via the oxidative stress pathway [68].

### 5.4. Diabetes Mellitus Diagnosis via Infrared Spectroscopy of Saliva: A Non-Invasive and Reliable Approach

Diabetes mellitus is one of the top five noncommunicable diseases that kill the most people worldwide, according to the WHO, with type 2 diabetes (DM2) being the most common [69]. Blood glucose monitoring is an invasive, painful, and expensive procedure. Several studies have demonstrated the potential of IR spectroscopy in the detection of diabetes mellitus through saliva analysis (Table 1). DM frequently reduces salivary flow, alters salivary protein expression, and increases salivary glucose levels [70,71,72]. Specific salivary biomarkers for DM, such as glucose, alpha-amylase, ghrelin appetite hormone, immunoglobulins, glycated end products, myeloperoxidases, and other markers of oxidative stress (e.g., salivary peroxidase) have been identified [3,73,74,75].

FTIR spectroscopy was used to analyze saliva samples from individuals with diabetes mellitus and healthy controls. In a first study of 1040 patients with 540 type 2 diabetics and 500 control subjects, the authors proposed a novel approach to diagnose DM2 based on saliva analysis using ATR-FTIR spectroscopy. The objective was to establish a strong foundation for future studies that can utilize this dataset to suggest alternative methods to the current gold standard for non-invasive diagnosis and management of DM2. They collected and measured the IR spectra of saliva samples from diabetic and healthy individuals and performed a statistical analysis to distinguish between them. They showed that ATR-FTIR spectroscopy could accurately detect diabetes mellitus using saliva samples in a non-invasive way and provided raw and processed data files and R scripts as supplementary materials for future research [15]. Another cross-sectional research using 23 patients’ saliva samples (2 healthy controls, 9 patients with diabetes mellitus, and 12 patients with both diabetes mellitus and periodontitis) showed that the fingerprint region between 600 and 1300 cm^−^^1^ (including the peak at band centered at 1076 cm^−^^1^ corresponding to the vibrational mode of skeletal cis conformation of DNA), 1403 cm^−^^1^ band of symmetric CH_3_ bending modes of protein methyl groups and symmetric CH_3_ bending of collagen, and 1451 cm^−^^1^ band of asymmetric CH_3_ bending modes of protein methyl groups may differ between control and diabetic patients [76].

In an animal study evaluating the saliva of normoglycemic, diabetic, and insulin-treated diabetic rats using ATR-FTIR spectroscopy, diabetic rats were classified with a sensitivity of 100% and an average specificity of 93.33% and 100%, respectively, using bands at 1452 cm^−^^1^ and 836 cm^−^^1^, respectively. The 1452 cm^−^^1^ (area for asymmetric CH_3_ bending modes of protein methyl groups) and 836 cm^−^^1^ (C_2_ endo/anti-B-form helix conformation) spectral bands were robust spectral biomarkers that were highly correlated with glycemia (R^2^: 0.801 and 0.788, respectively; p < 0.01). It is critical to remember that the C_2_ conformation of sugars at 836 cm^−^^1^ does not indicate the presence of glucose. The aldehyde structure for the conversion of glucose into a cyclic hemiacetal (glucopyranose) occurs at the C_4_-C_5_ bond [77], resulting in a peak at 1375 cm^−^^1^ [78]. In ROC analysis, these salivary spectral bands demonstrated 100% sensitivity and 100% specificity. Both PCA-LDA and hierarchical cluster analysis (HCA) classifications achieved an accuracy of 95.2% in discriminating non-diabetic, diabetic, and insulin-treated diabetic rats based on their salivary spectra. The ability of these two salivary ATR-FTIR bands to distinguish and differentiate indicates that they may serve as diagnostic and monitoring tools for DM. Insulin treatment reversed the salivary spectra observed in patients with hyperglycemia. The identification of salivary biomarkers using univariate and multivariate analyses offers a promising and environmentally friendly approach for diabetes monitoring [79]. This method allows for the analysis of other molecules in addition to glucose, which could indicate metabolic control in saliva. Although glucose is the primary molecule for assessing metabolic control in the blood, the discovery of glucose transporters in salivary ductal cells highlights the potential of salivary analysis for evaluating other biomarkers. Overall, IR analysis of salivary biomarkers could provide a new, reliable, and non-invasive option for diabetes monitoring. IR spectroscopy can highlight the molecular changes associated with the development and progression of diabetic complications, such as diabetic retinopathy, neuropathy, or nephropathy. This information can aid in personalized risk assessment, allowing for early detection and intervention to prevent or mitigate complications. Moreover, IR spectroscopy can be used to assess nutritional status and guide personalized dietary recommendations for individuals with diabetes mellitus [80]. 

### 5.5. Real-time Drug Monitoring Using Infrared Spectroscopy of Saliva: A Promising Approach for Personalized Medicine

IR spectroscopy can be used to analyze the composition of saliva and detect changes associated with drug metabolism. Drugs can affect the levels of certain biomolecules in the saliva, including proteins and lipids, which can be detected using IR spectroscopy. In addition, this technique can be used to monitor drug levels in saliva. Several studies have demonstrated the potential of IR spectroscopy for drug monitoring using salivary analysis (Table 1). 

Saliva can contain traces of heroin for a few hours after consumption. When heroin is smoked, the concentration of the drug can reach 20 mg/mL in the first few minutes following administration [81]. Heroin displays distinctive absorbance characteristics in the range of 1800–750 cm^−^^1^ [82]. Within the range of 1700–1000 cm^−^^1^, codeine exhibits characteristic absorbance properties [83]. Spectroscopy can simultaneously detect various cocaine metabolites owing to their similar absorption ranges. Studies indicate that the 1710–1800 cm^−^^1^ spectral region is ideal for detecting cocaine, even in the presence of commonly consumed substances such as diluents, adulterants, medications, and drinks. Although mouthwash and alcohol consumption affect saliva spectra, they do not significantly interfere with the critical cocaine absorption range. By using the dried sample technique to minimize water absorption, cocaine can be detected in saliva at a limit of 0.02 mg/mL without the need for additional sample preparation. Although this limit is sufficient for real-life scenarios, it can be lowered by using a quantum cascade laser, a higher responsivity detector, and an improved interaction zone design between the laser beam and sample to increase the evanescent field absorption. This non-invasive and straightforward technique could be an effective means of detecting cocaine use [84].

### 5.6. Early Detection of Chronic Kidney Disease using Infrared Spectroscopy Analysis of Saliva

Chronic kidney disease (CKD) can be difficult to diagnose in its early stages because the symptoms are often nonspecific or may not be present. At present, the clinical detection of CKD involves a persistent elevation in the urinary albumin excretion rate and/or a reduction in the glomerular filtration rate. Consequently, there is a need for the development of cost-effective, non-invasive, and accurate diagnostic platforms for CKD. A study involving 14 patients with CKD and 14 healthy control subjects matched for age and gender was conducted to compare salivary components using ATR-FTIR spectroscopy. The study identified several salivary components, of which four exhibited significantly different expression (*p* < 0.05) between CKD patients and control subjects. Among these, thiocyanate (SCN^−^, 2052 cm^−^^1^, C-N stretching) and the vibrational modes of phospholipids/carbohydrates (924 cm^−^^1^, C-O, C-C stretching, C-O-H, and C-O-C deformation) using the original and second-derivative spectra via ATR-FTIR have the potential to serve as salivary biomarkers for distinguishing CKD patients from healthy subjects [85]. SCN^−^ is a common pseudohalide thiolate, which is small and acidic in nature and is present in various extracellular fluids, such as saliva and plasma. It is synthesized from cyanide either by mitochondrial rhodanese or by entering the bloodstream through the diet [86]. Patients with CKD exhibit elevated levels of plasma SCN^−^ due to a reduction in kidney elimination [87]. The concentration of SCN^−^ in saliva is primarily dependent on the active transcellular transport of SCN^−^ in acinar cells rather than on the plasma concentration of SCN^−^ [86]. The combination of the original and second-derivative spectra by ATR-FTIR of the 924 cm^−^^1^ vibrational modes demonstrated 92.8% sensitivity and 85.7% specificity for CKD detection with an AUC of 0.88. The pathophysiological effects of this wavenumber, which are associated with phospholipids and carbohydrates, are not fully understood. However, despite the inability to identify the exact molecule, the 924 cm^−^^1^ peak in the second derivative ATR-FTIR spectra has clear potential as a diagnostic tool [85]. IR spectroscopy can contribute to personalized medicine for CKD by providing valuable information about kidney function, metabolic changes, and disease progression based on specific spectral features (Table 1). IR spectroscopy should be used in conjunction with existing diagnostic tools and clinical evaluations to ensure an accurate diagnosis and appropriate treatment decisions.

**Table 1:** Overview of the most important wavenumber regions, assigned compound classes, assigned bands, and suggested pathophysiological biomarkers/function.

Pathology	Most Important Wavenumber Regions	Compound Class	Band Assignment	Suggested Pathophysiological Biomarker/Function	References
Dental caries	2150–1950 cm^−1^	Thiocyanate	N=C=S stretching	Antibacterial properties	[28,29,30,31]
	1765–1725 cm^−1^	Esters, lipids, and carbohydrates	C=O stretching		[28,29,30,31]
	1700–1590 cm^−1^	Protein	C=O stretching (Amide I) C-N stretching (Amide I)	Pathological microflora	[28,29,30,31]
	1590–1505 cm^−1^	Protein	N-H in-plane bending (Amide II)	Pathological microflora	[28,29,30,31]
	1430–1360 cm^−1^	Carbon-phosphate	C=O and CH_2_/CH_3_ bonds		[28,29,30,31]
	1078–900 cm^−1^	Phosphate	PO_2_^−^-stretching		[28,29,30,31]
Periodontitis	2800–3000 cm^−1^	Lipids	CH_2_ and CH_3_ stretching	Lipid oxidation	[34,35]
	1713 cm^−1^	Lipids	C=O stretching		[34]
	1652 cm^−1^	Protein	C=O stretching (Amide I)		[34]
	1230 to 1180 cm^−1^	Phosphate	PO_2_-stretching	Base-pared DNA strand	[33]
	950–1080 cm^−1^	Carbohydrates	CO-O-C- stretching	Glycosylated proteins (α-amylase)	[36]
Oral cancer	2924 and 2854 cm^−1^	Membranous lipids	Asymmetric and symmetric C-H stretching of CH_2_ and CH_3_ methylene groups	Fatty acids within cellular membranes	[37]
	1543 cm^−1^	Transmembrane proteins	Amide II	α-helix	[37]
	1072 cm^−1^	Nucleic acids	Phosphate bonds	DNA	[37]
Salivary gland tumors	1664–1641 cm^−1^	Protein	C=O stretching (Amide I)	α-helix	[53]
	1648 cm^−1^	Protein	C=O stretching (Amide I) C-N stretching (Amide I) N-H bending (Amide I)	α-helix	[16,42,46,48,49,50,51,52]
	1631 cm^−1^	Protein	C=O stretching (Amide I) C=C stretching (Amide I)	β-sheet structure	[53]
	1543 cm^−1^	Protein	N-H bending (Amide II) C-N stretching (Amide II)		[16,42,46,48,49,50,51,52]
	1515 cm^−1^	Protein	Tyrosine ring	α-amylase, albumin, cystatins, mucins, proline-rich proteins, sIgA	[38]
	1315 cm^−1^	Protein	C-N stretching (Amide III) N-H bending (Amide III)	α-amylase, albumin, cystatins, mucins, proline-rich proteins, sIgA	[38]
	1000 to 1200 cm^−1^	Carbohydrates	C-O stretching	Glycosylated α-amylase, mucins or other sugar residues	[21,39,42]
	1119 cm^−1^	Carbohydrates	C-O stretching C–O–C-stretching	Glycosylated α-amylase, mucins or other sugar residues	[45,46,47]
	1078 cm^−1^	Phosphate	PO_2_^−^-stretching	Inorganic phosphates and phospholipids	[39,40]
Breast cancer	1433–1302.9 cm^−1^	Proteins and lipids	COO^−^ stretching		[55]
	1041 cm^−1^	Nucleic acids and glycogen	Symmetric PO_2_^−^stretching		[55]
COVID-19	1785–1729 cm^−1^	Lipids	C=O stretching C=C stretching		[57]
	1718–1705 cm^−1^	Protein	C=O stretching C-N stretching		[57]
	1680 cm^−1^	Protein	C=O stretching C-N stretching		[57]
	1600–1200 cm^−1^	Protein	Amide I, II and III		[57]
	1612–1606 cm^−1^	Nucleic acid	Adenine vibration in DNA		[57]
	1560–1464 cm^−1^	Protein	C=O stretching C-N stretching	IgG	[63]
	1429 cm^−1^	Nucleic acid	CH_2_-bending	RNA virus	[61]
	1220 cm^−1^	Nucleic acid	PO_2_-stretching	Host organism’s response to viral infection	[61]
	1084 cm^−1^	Nucleic acid	Symmetric PO_2_-stretching in nucleic acids	Host organism’s response to viral infection	[61]
	1069 cm^−1^	Nucleic acid	C-O stretching in ribose	Host organism’s response to viral infection	[61]
	1041 cm^−1^	Nucleic acid	Symmetric PO_2_-stretching in nucleic acids	Host organism’s response to viral infection	[61]
	1025–1021 cm^−1^	Carbohydrates	C-O stretching		[57]
	961 cm^−1^	Nucleic acid	Desoxyribose		[57]
	930–909 cm^−1^	Nucleic acid	PO_2_-stretching		[57]
Neonatal sepsis	1640 cm^−1^	Protein	C=O stretching (Amide I) N-H bending (Amide I)	Changes in protein linked to inflammatory process	[67]
	1545 cm^−1^	Protein	C-N stretching (Amide II) N-H bending (Amide II)	Changes in protein linked to inflammatory process	[67]
	1301 cm^−1^	Protein	C-H stretching (Amide III) N-H bending (Amide III)	Changes in protein linked to inflammatory process	[67]
	1240 cm^−1^	Protein	C-N stretching (Amide III) N-H bending (Amide III)	Changes in protein linked to inflammatory process	[67]
	1051 cm^−1^	Nucleic acid	C-O stretching	Changes in DNA linked to inflammatory process	[67]
	1037 cm^−1^	Nucleic acid	C-O stretching	Changes in DNA linked to inflammatory process	[67]
	970 cm^−1^	Nucleic acid	C-O stretching	Changes in DNA linked to inflammatory process	[67]
Diabetes mellitus	1452 cm^−1^	Protein	Asymmetric CH_3_ bending	High correlation with glycemia	[77]
	1451 cm^−1^	Protein	Asymmetric CH_3_ bending		[76]
	1403 cm^−1^	Protein	Symmetric CH_3_ bending Symmetric CH_3_ bending		[76]
	1076 cm^−1^	Nucleic acid	Skeletal cis conformation of DNA		[76]
	836 cm^−1^	Carbohydrates	C_2_ endo/anti-B-form helix conformation of sugar	High correlation with glycemia	[77]
Chronic kidney disease	2052 cm^−1^	Thiocyanate	C-N stretching	Increased SCN^−^ concentration in plasma transported to the saliva via acinar cells by active transcellular transport	[85]
	924 cm^−1^	Phospholipids carbohydrates	C-O stretching C-C stretching C-O-H deformation C-O-C deformation	Unknown	[85]

## 6. Conclusions

In the future, we expect to see further advancements in IR spectroscopy, which will enhance its utility in salivary analysis. Salivary analysis with IR spectroscopy is a powerful tool in personalized medicine, as it allows for non-invasive and accurate analysis of a patient’s chemical composition, providing valuable insights into their health status and enabling personalized treatment plans. By analyzing the chemical composition of saliva using IR spectroscopy, personalized medicine can create individualized treatment plans for patients. For example, patients with DM may have specific salivary biomarkers that indicate poor glycemic control. Salivary analysis using IR spectroscopy can detect these biomarkers, enabling personalized treatment plans that may include changes in diet, exercise, and medication. This is also valuable for drug development and personalized drug therapy. IR spectroscopy can be used to identify the chemical composition of drugs and their metabolites in saliva, allowing the development of personalized drug therapies based on a patient’s unique metabolic profile. It is important to note that while IR spectroscopy holds promise in personalized medicine, further research, validation, and standardization are necessary to establish its clinical utility across different stages and subtypes of the disease.

Further investigations involving a larger number of patients, including those with complex diseases, are necessary to establish a comprehensive methodology that accounts for the diversity of saliva in the human population. The development of standardized protocols for sample collection, preparation, and analysis can further enhance the utility of salivary diagnostics and pave the way for personalized medicine. 

Another area of development is the use of portable IR spectrometers that can be used in point-of-care testing. These devices can be used for the rapid and non-invasive diagnosis of diseases using saliva samples, making them ideal for use in clinical settings. There is ongoing research on the use of new IR spectroscopy techniques such as surface-enhanced infrared absorption (SEIRA) spectroscopy and sum frequency generation (SFG) spectroscopy. These techniques can provide higher sensitivity and specificity for the analysis of salivary biomolecules, allowing the detection of diseases at an earlier stage. Overall, the use of IR spectroscopy for salivary analysis is promising. With continued research and development, we expect to see further advancements in IR spectroscopy techniques that will enable a more accurate and rapid diagnosis of various diseases using saliva samples. Moreover, it has the potential to revolutionize disease treatment, thereby rendering personalized medicine a reality for many patients.

## Data Availability

Not applicable.
